# Developmental stage of oligodendrocytes determines their response to activated microglia *in vitro*

**DOI:** 10.1186/1742-2094-4-28

**Published:** 2007-11-26

**Authors:** Brandon A Miller, Jeannine M Crum, C Amy Tovar, Adam R Ferguson, Jacqueline C Bresnahan, Michael S Beattie

**Affiliations:** 1 Brain and Spinal Injury Center, Department of Neurological Surgery, University of California San Francisco, 1001 Potrero Ave, Building 1, Room 101, San Francisco, CA 94143, USA; 2 Department of Neuroscience, The Ohio State University, 4140 Graves Hall 333 W. 10^th ^Ave., Columbus, OH 43210, USA

## Abstract

**Background:**

Oligodendrocyte progenitor cells (OPCs) and mature oligodendrocytes are both lost in central nervous system injury and disease. Activated microglia may play a role in OPC and oligodendrocyte loss or replacement, but it is not clear how the responses of OPCs and oligodendrocytes to activated microglia differ.

**Methods:**

OPCs and microglia were isolated from rat cortex. OPCs were induced to differentiate into oligodendrocytes with thyroid hormone in defined medium. For selected experiments, microglia were added to OPC or oligodendrocyte cultures. Lipopolysaccharide was used to activate microglia and microglial activation was confirmed by TNFα ELISA. Cell survival was assessed with immunocytochemistry and cell counts. OPC proliferation and oligodendrocyte apoptosis were also assessed.

**Results:**

OPCs and oligodendrocytes displayed phenotypes representative of immature and mature oligodendrocytes, respectively. Activated microglia reduced OPC survival, but increased survival and reduced apoptosis of mature oligodendrocytes. Activated microglia also underwent cell death themselves.

**Conclusion:**

Activated microglia may have divergent effects on OPCs and mature oligodendrocytes, reducing OPC survival and increasing mature oligodendrocyte survival. This may be of importance because activated microglia are present in several disease states where both OPCs and mature oligodendrocytes are also reacting to injury. Activated microglia may simultaneously have deleterious and helpful effects on different cells after central nervous system injury.

## Background

Oligodendrocytes develop from a bipotential progenitor cell, often referred to as an oligodendrocyte progenitor cell (OPC), or oligodendrocyte – type 2 astrocyte cell (O2A), as it can differentiate into either an oligodendrocyte or astrocyte *in vitro *[1]. Though more prevalent in the immature CNS, OPCs persist in the CNS of mature animals and humans [2,3], and have been shown to respond to CNS injury by proliferating and possibly taking the place of mature oligodendrocytes that are lost during injury or disease [4-6].

Both OPCs and oligodendrocytes die in a variety of CNS diseases. In periventricular leukomalacia (PVL), OPCs are lost as a consequence of hypoxia, ischemia, or intrauterine infection [7]. This loss of OPCs, and the resulting failure to replace mature oligodendrocytes, is thought to be the pathologic cause of spastic cerebral palsy [8]. Mature oligodendrocytes are lost in multiple sclerosis (MS), the most common disease of the adult CNS, affecting over 250,000 people living in the US [9]. Oligodendrocytes and OPCs also die after CNS trauma, such as brain and spinal cord injury [10,11] and it has been shown that oligodendrocyte apoptosis in experimental spinal cord injury peaks over a week after the initial insult [12,13]. This delayed oligodendrocyte death may reduce the effectiveness of neural conduction in the spared axons that often exist after spinal cord injury.

CNS inflammation occurs in both disease and trauma, and is mediated in part by microglia, the resident immune cells of the CNS. Microglia originate from bone marrow and migrate into the CNS during early stages of development [14]. Microglia display graded levels of activation in the CNS, from resting, highly ramified microglia, to phagocytic macrophages [15]. Microglia react quickly in response to CNS injury or disease [16], migrating into an injury site [17] and secreting a wide array of molecules that can be toxic to OPCs and oligodendrocytes, including tumor necrosis factor-α (TNFα) [18-20], glutamate [21], and free radicals [22]. Activated microglia contribute to OPC and oligodendrocyte death in models of PVL and MS [23-25]. Furthermore, molecules that induce oligodendrocyte death can also lead to microglial activation, such as glutamate [26,27] and proinflammatory cytokines [28].

*In vitro*, microglia are capable of inducing OPC death even without the two cell populations being in direct contact [29]. However, *in vivo *microglia have been observed in close proximity to dying oligodendrocytes after spinal cord injury [13]. This proximity after injury suggests a mechanism by which microglia may influence oligodendrocyte and OPC survival, as it has been shown *in vitro *that microglia in contact with oligodendrocytes can induce oligodendrocyte death via membrane-bound TNFα which is more potent than soluble TNFα [30]. Additionally, any soluble factors secreted by microglia could have a higher effective concentration if secreted into a small space between cells.

There is also evidence that microglia may play a protective or helpful role in the injured CNS [31,32]. *In vitro*, microglia can be recruited by soluble factors released by stressed oligodendrocytes, and support oligodendrocyte survival via insulin-like growth factor 2 [33,34]. Additionally, cytokines produced by microglia may aid in repair after injury, as mice lacking TNFα undergo delayed remyelination [35]. Even the observations of Shuman and colleagues [13], that activated microglia are found in contact with apoptotic oligodendrocytes after spinal cord injury, raises the question of whether microglia destroy oligodendrocytes that would otherwise survive after injury, or are simply phagocytosing oligodendrocytes already destroyed by other toxins in the damaged CNS. Some data suggest that microglia play a dual role in CNS injury, exacerbating damage in some instances or at some times, and promoting repair or regeneration at others [36,37]. Shuman and colleagues [13] also reported that microglia undergo apoptosis after spinal cord injury. It has been demonstrated that certain types of toxin-induced microglial activation can result in microglial death both *in vitro *and *in vivo *[38,39] and microglial death has also been described in concert with microglial activation in other *in vivo *injury paradigms [40].

**Figure 1:**
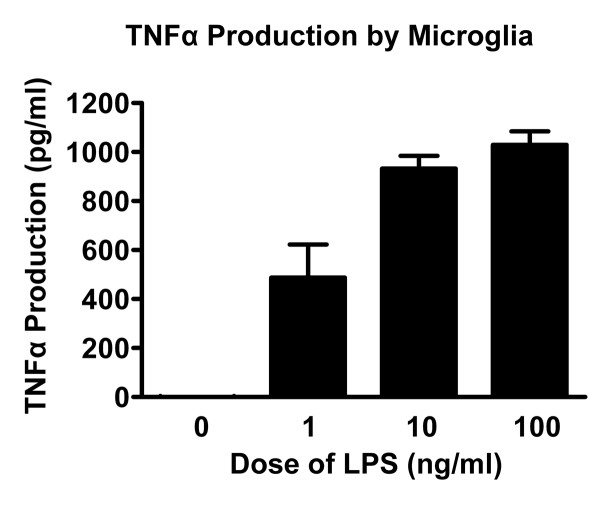
**TNFα production by microglia**. In a preliminary experiment, microglia alone were treated with LPS at varying doses and TNFα production was assessed 24 hours later. LPS induced TNFα production in a dose dependent manner.

The current studies were carried out to better to determine the effect of activated microglia on oligodendrocytes at different developmental stages and to assess microglial survival after activation. Though many studies have examined OPC and oligodendrocyte response to activated microglia, no study, to our knowledge, has directly compared the response of OPCs and oligodendrocytes to activated microglia under identical culture conditions. Examining OPC and oligodendrocyte survival under identical conditions is important, as these cell types are both present together in the injured CNS and may respond differently to the effects of microglial activation. In this study, we utilized lipopolysaccharide (LPS) to activate microglia. LPS activates Toll-like receptor 4 and causes microglia to release proinflammatory cytokines and become phagocytic [41,42]. Studies in our laboratory showed that LPS induced TNFα release from microglia in a dose-dependent fashion (Fig. 1). We found that LPS-activated, but not non-activated, microglia reduced OPC survival. However, both LPS-activated and non-activated microglia increased mature oligodendrocyte survival even as microglia themselves underwent activation-induced cell death. These findings suggest that oligodendrocytes at different developmental stages respond differently to activated microglia and that OPCs and mature oligodendrocytes may undergo different fates in the face of microglial activation *in vivo*.

## Methods

### Cell culture

All procedures were approved by The Ohio State University Institutional Laboratory Animal Care and Use Committee. All reagents were purchased from Sigma (St. Louis, MO) unless otherwise noted. OPCs and oligodendrocytes were isolated by a protocol similar to that described by McCarthy and de Vellis [43] with some modifications. Postnatal day 2 Long Evans rat pups were sacrificed by rapid decapitation and the cortices were removed and placed in media containing 7.1 mg/ml NaCl, 0.36 mg/ml KCl, 0.166 mg/ml KH_2_PO_4_, 2.57 mg/ml d-Glucose, 2.4 mg/ml NaHCO_3_, 0.01 mg/ml phenol red, 0.9 mg/ml BSA Fraction V, and 0.33 mg/ml MgSO_4_, (hereafter referred to as "dissection media") and minced. The tissue was resuspended with 10 mls of dissection media with 0.280 μg/ml trypsin and shaken on an orbital shaker at 200 rpm for 20 minutes, at 37°C. Next, 10 mls of dissection media with 1.28 μg/ml DNAse and 83.2 μg/ml soy bean trypsin inhibitor was added and the mixture was briefly shaken and centrifuged at 690 rcf for 5 minutes. The pellet was then resuspended in 3 ml of dissection media with 80 μg/ml DNAse and 520 μg/ml soy bean trypsin inhibitor and triturated to a single cell suspension. Cells were resuspended in DMEM (Invitrogen, Carlsbad, CA) with 10% fetal bovine serum containing 292 μg/ml L-glutamine, 1 mM sodium pyruvate (Invitrogen) and 0.04 mg/ml gentamicin (Invitrogen) at a volume of approximately 5 ml per brain. The suspension was then plated into uncoated T-75 flasks at 10 mls per flask. Flasks were kept in an incubator at 37°C and 5% CO_2_. Media was replaced after 24 hours and every 4–6 days thereafter.

Cells were allowed to proliferate until confluent, corresponding to day 9–13 post-dissection. At this time, the mixed cortical cultures consisted of a layer of astrocytes adherent to the bottom of the flasks, with OPCs and microglia growing on top of the astrocytes. Before isolating pure OPCs, the numbers of microglia in the flasks were reduced by placing sealed flasks on an orbital shaker at 200 rpm for 2 hours. After shaking, the supernatant was replaced with fresh media and flasks were returned to the incubator for 2 hours. After equilibration, flasks were shaken overnight to detach OPCs from the microglia-depleted population. At the completion of the overnight shake, floating cells were resuspended in 4 ml of serum-free DMEM Sato with either thyroid hormone (as we have described previously, [44]) in order to induce maturation of OPCs to oligodendrocytes, or 10 ng/ml PDGF and 10 ng/ml bFGF in order to maintain cells as OPCs. In order to remove any microglia that were not removed during the 2 hour shake, the cell suspension was plated onto a sterile petri dish with a radius of 2.5 mm and returned to the incubator for 45 minutes where both OPCs and microglia adhered to the petri dish. Petri dishes were then gently rinsed with culture media in order to remove OPCs, but leave the more tightly adherent microglia behind. The resulting cell suspension was adjusted to a density of 20,000 cells per ml and plated onto poly-L-lysine coated 12 mm glass coverslips in 4-well plates, with 500 ul of cell suspension added per well. These OPC/oligodendrocyte cultures were >95% pure with <5% microglia as verified by immunocytochemistry.

### Addition of microglia to oligodendrocyte cultures and experimental timeline

In selected experiments, microglia were added to OPCs or oligodendrocytes exactly one day after OPCs/oligodendrocytes were isolated from flasks containing mixed glial cultures. LPS treatments took place exactly 2 days after OPC/oligodendrocyte plating, and fixation took place exactly 3 days after OPC/oligodendrocyte plating. Microglia were harvested from flasks derived from the same primary dissections as the OPCs or oligodendrocytes with which they were combined. To remove microglia from flasks without removing OPCs, T75 flasks were tapped on the lab bench and the supernatant removed. Microglia were resuspended in the same medium as the OPCs or oligodendrocytes with which they were combined. When plated alone, the resuspended cells were shown to be > 95% pure microglia. Microglia were plated onto pre-existing OPC or oligodendrocyte cultures at a ratio of 1:1 by initially resuspending microglia at twice the desired concentration, removing 250 μl of media from the OPC or oligodendrocyte cultures and adding 250 μl of microglia suspension to OPC or oligodendrocyte cultures. The 1:1 ratio of microglia to oligodendrocyte lineage cells was chosen to be close to that observed in counts of microglia:oligodendrocyte ratios in the rat dorsal column (~2:3, data not shown).

### Immunocytochemistry and cell counts

At the conclusion of all experiments, cells were fixed with 2% formaldehyde prepared from paraformaldehyde. All primary antibodies were purchased from Chemicon (Temecula, CA) unless otherwise noted. All secondary antibodies were purchased from Molecular Probes (Carlsbad, CA). Antibodies were diluted in Hank's Balanced Salt Solution with 5% donor calf serum and coverslips were rinsed twice between all antibody applications. For NG2 labeling, cells were first permeabilized with 0.1% Triton for 10 minutes, and then incubated with anti-NG2 antibody at a concentration of 1:400 for 1.5 hours, followed by goat anti-rabbit Alexa 594 at 1:100 for 1 hour. For A2B5 labeling, cells were incubated with an antibody to A2B5 conjugated to Alexa 488 (Chemicon) at 1:100 for 1.5 hours. For GalC labeling, cells were incubated with an antibody to GalC at 1:400 for 1 hour followed by goat anti-mouse Alexa 488 at 1:200 for 1 hour. For MBP labeling, cells were first permeabilized with 0.1% Triton for 10 minutes and incubated with an antibody against MBP at 1:200 for 1 hour followed by goat anti-rabbit Alexa 488 at 1:100 for 1 hour. For IB4 labeling, which was completed following staining for OPC/oligodendrocyte markers, cells were incubated with Alexa 594-conjugated IB4 (Molecular Probes) at 1:100 for 30 minutes in a solution of HBSS with 1 mM calcium and no serum. For all experiments, cells were incubated with 10 μg/ml Hoechst for 5 minutes and mounted on glass slides with Immu-Mount (Thermo, Pittsburgh, PA) after antibody application.

Cell counts were conducted by an experimenter who was blind to experimental conditions, using a Zeiss Axioplan 2 microscope (Carl Zeiss, Thornwood, NY). Counts were conducted using the 40× objective. Continuous, non-overlapping fields were assessed across the entire coverslip diameter and every cell whose cell body was within the eyepiece reticle was assessed. For experiments involving OPCs or oligodendrocytes, the control condition was defined as OPCs or oligodendrocytes alone with no microglia or LPS added. For mature oligodendrocytes, both live and dead cells were visible, with live cells having intact nuclei and cell bodies, and dead cells having absent or fragmented nuclei and fragmented cell bodies. Counts of both live and dead oligodendrocytes were performed and values are expressed as percent cell death. Actual cell counts rather than biochemical assays of cell death were used to allow discrimination of microglial and oligodendrocyte/OPC cell death in the combined culture studies. Previous work in our laboratory has shown that morphology-based live/dead counts of oligodendrocyte lineage cells correlate well with lactate dehydrogenase (LDH) assay-based cell death estimates (data not shown). Live OPCs were defined as A2B5 positive cells with two or more intact processes and intact nuclei. It was not possible to reliably quantify dead OPCs, as dead OPC cell bodies were often absent and it could not be determined how many OPCs were represented by debris, composed of OPC processes, left behind after fixation and staining. Therefore, for OPCs, values were expressed as live cells counted relative to control. For microglia, live cells were visible as IB4 stained cells with intact nuclei, and values were expressed relative to those under conditions in which microglia, but not LPS, were added to OPC/oligodendrocyte cultures.

### Caspase assay

A commercially available caspase assay (FLICA, Immunochemistry Technologies, Bloomington, Minnesota) that utilizes a fluorescently-tagged poly caspase inhibitor was utilized in accordance with the manufacturer's recommendations. The use of this assay has been described previously by Grabarek and colleagues [45]. Caspase positive oligodendrocytes were defined as cells with fragmented nuclei that also labeled brightly with the caspase indicator, so that the cell body and processes were visible to confirm oligodendrocyte morphology. Cells counts were performed as described above, and caspase activation was expressed as percent of control.

### TNFα ELISA

A TNFα ELISA was used to verify that the selected dose of LPS activated microglia under different media conditions. TNFα concentrations were determined using a double sandwich ELISA following R&D Systems' (Minneapolis, MN) recommended protocol. Briefly, 96 well plates were coated with 4 μg/ml of capture antibody (R&D Systems) in PBS pH 7.4 overnight. Nonspecific binding was blocked with PBS containing 1% bovine serum albumin (BSA) for one hour. Experimental samples and known standards were diluted 1:2 in PBS with 0.05% Tween and 0.1% BSA and added to the capture antibody for 2 hours at room temperature. 100 μl per well of biotinylated anti-rat TNFα antibody (R&D Systems) at a concentration of 400 ng/ml was added for 2 hours at room temperature followed by streptavidin horseradish peroxidase conjugate at 1:4000 for 20 minutes. 100 μl per well of K-Blue Max substrate (Neogen, Randolph, WI) was added and allowed to develop in the dark for 20 minutes. The reaction was stopped by the addition of 1 M H_2_SO_4_. Plates were read using a Genios plate reader (Tecan, Switzerland) at 490 nm.

### Cell proliferation assay

A BrdU incorporation assay, similar to that employed by Kondo and Raff [46] was used to assess OPC and oligodendrocyte proliferation. Cells were exposed to 20 uM BrdU for six hours prior to fixation, and then fixed in 2% formaldehyde as previously described. Cells were post-fixed in 100% methanol for 10 minutes at -20°C, followed by DNA denaturation with 2 M hydrochloric acid for 30 minutes. Acid was then neutralized with 0.1 M sodium borate at pH of 8.5 for 10 minutes. Cells were then permeabilized with 0.1% Triton for 10 minutes and incubated with primary antibody against BrdU (hybridoma monoclonal antibody developed by Stephen J. Kaufman, Developmental Studies Hybridoma Bank, The University of Iowa, Department of Biological Sciences, Iowa City, IA) at 1:5 for 1 hour followed by goat anti-mouse secondary antibody conjugated to Alexa 594 at 1:100 for 1 hour. This staining was done in combination with A2B5 labeling for OPCs and followed by Hoechst labeling of nuclei. BrdU labeling was evaluated by examining five random fields per coverslip and the percentage of OPCs or oligodendrocytes incorporating BrdU was compared to that of the control condition. All BrdU, cell survival, and caspase experiments were replicated using cells from at least two different primary cultures, and all "n" values refer to individual coverslips on which experiments were carried out. OPC/oligodendrocyte survival, BrdU and caspase experiments were analyzed via factorial ANOVA. Additionally, a one way ANOVA with Tukey's post-hoc test was conducted to determine what groups differed from OPCs or oligodendrocytes alone. All error bars represent ± SEM. Significance was set at p < 0.05.

### LDH assay for assessment of microglia death

A commercially available LDH assay kit (Roche, Indianapolis, IN) was used to quantify LDH release in experiments with microglia grown alone. In 3 independent replications, microglia were isolated as described above, and plated at concentrations varying from 50,000 to 150,000 cells/ml into a 96 well plate coated with poly-L-lysine. 24 hours after plating, 10 ng/ml LPS was administered for 24 hours and LDH release was assayed according to the manufacturer's instructions. For positive controls, microglia were treated with 1% triton for 10 minutes prior to LDH release quantification. Cell death was measured as [experimental value - negative control]/[positive control - negative control]. LDH release by microglia treated in the two conditions was compared using Student's T-test.

## Results

### OPCs cultured with bFGF and PDGF express markers of both immature and mature oligodendrocyte lineage cells

**Figure 2:**
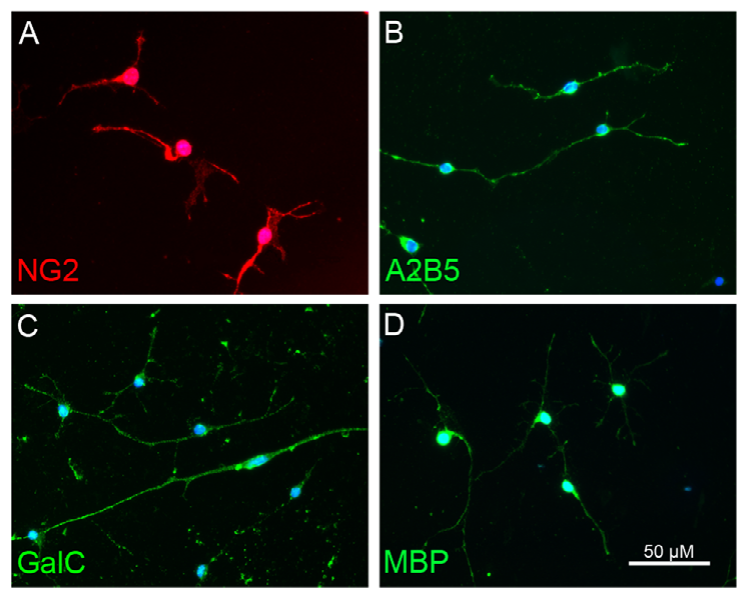
**OPC morphology**. OPCs grown in media containing bFGF/PDGF labeled for oligodendrocyte markers at day 3 *in vitro*. OPCs labeled positively for NG2 (A), A2B5 (B), GalC (C) and MBP (D).

OPCs grown continuously with PDGF and bFGF were observed under phase contrast microscopy to have a bipolar morphology typical of OPCs by day 1 *in vitro*. On day 3 *in vitro*, cells were labeled with the OPC markers NG2 and A2B5 in their somata and processes (Fig. 2A, B). OPCs also stained positively for GalC and faintly for MBP, typically considered to be markers of mature oligodendrocyte lineage cells (Fig. 2C,D). These observations suggest that the OPCs in this *in vitro *system were beginning the transition toward mature oligodendrocytes [47].

### LPS-activated microglia release TNFα

A preliminary experiment was conducted to determine the response of microglia to varying doses of LPS. Microglia were exposed to 1 ng/ml, 10 ng/ml and 100 ng/ml of TNFα for 24 hours. This resulted in increasing production of TNFα (Fig. 1). There was no detectable TNFα production by OPCs or mature oligodendrocytes alone treated with LPS or microglia cultures that were not treated with LPS (data not shown). We chose 10 ng/ml LPS for use in further experiments, as this dose has been shown by others to induce microglia-mediated OPC death [48] and was in the middle of our range of LPS doses that induced TNFα production. In microglia isolated for use in OPC/oligodendrocyte survival experiments, there was no significant effect of media composition (OPC versus oligodendrocyte media) on TNFα production by microglia (data not shown).

### LPS-activated microglia reduce OPC survival

**Figure 3:**
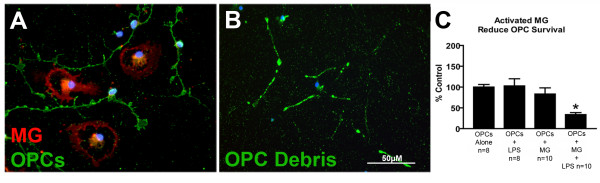
**LPS-activated microglia induce OPC cell death**. OPCs were cultured in combination with microglia (A). Dead OPCs were not able to be quantified due to scattered debris (B), so OPC survival was measured by counting the number of live OPCs. LPS or microglia alone had no effect on OPC survival but microglia activated with LPS significantly reduced OPC survival (C, * = p < 0.05 from control, Tukey's, error bars = SEM).

For these experiments, pure OPC cultures, without and with microglia added, were utilized (Fig. 3A). There was no effect of 10 ng/ml LPS on the number of live OPCs when OPCs were cultured alone; additionally, there was no effect of microglia alone on OPC number. In OPC-microglia combined cultures, there was a significant reduction in OPC number when 10 ng/ml LPS was added for 24 hours (Fig. 3B,C). The number of OPCs in the presence of microglia plus LPS was reduced to 34 ± 5% of control values (p = 0.002, ANOVA, Tukey's post-hoc), indicating that LPS-activated microglia decreased OPC survival. Factorial ANOVA revealed a significant (p = 0.036) interaction between microglia and LPS, and a significant main effect of microglia (p = 0.001).

### Effects of activated microglia on proliferation of OPCs

**Figure 4:**
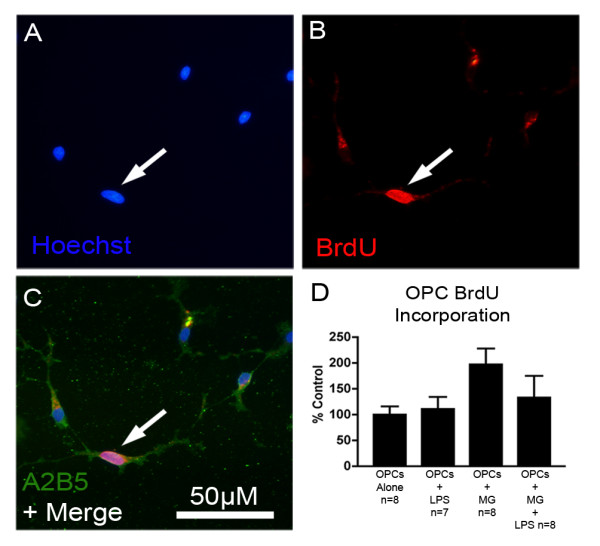
**OPC proliferation as measured by BrdU incorporation**. To assure that reduced OPC survival in the presence of LPS-activated microglia was not due to a reduction in OPC proliferation, a BrdU incorporation study was conducted. Some OPC nuclei (A) also incorporated BrdU (B). OPCs were also labeled with antibody to A2B5 (C) to confirm that BrdU positive cells were OPCs. OPC proliferation, as assessed by cell counts, was not altered by microglia or LPS-activated microglia (though there was a trend toward increased OPC proliferation in the presence of microglia, p = 0.056, factorial ANOVA for main effect of microglia) verifying that lower OPC numbers were due to OPC loss rather than reduced OPC proliferation (D, error bars = SEM).

To determine if the reduction of OPC number by activated microglia was due to a decrease in OPC proliferation, a BrdU assay was performed. OPCs were observed to incorporate BrdU in all experimental conditions, with an average 6% of OPCs incorporating BrdU in the control condition (Fig. 4A,B,C). There were no significant main effects or interactions between 10 ng/ml LPS or microglia on OPC proliferation (Fig. 4D), however the main effect of microglia on increasing OPC proliferation approached statistical significance (p = 0.056). This verifies that reduced OPC numbers due to microglia activation were due to OPC loss and could not be due to decreased OPC proliferation.

### Oligodendrocytes cultured with thyroid hormone only express markers of mature oligodendrocytes

**Figure 5:**
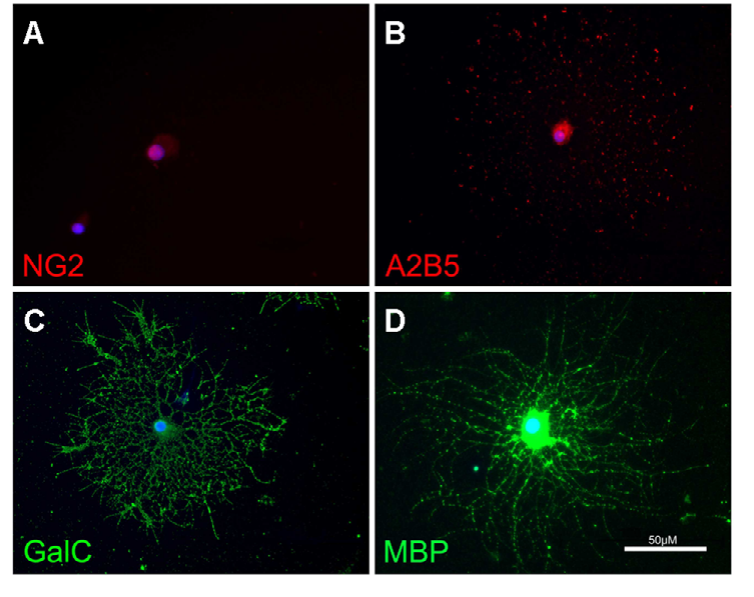
**Oligodendrocyte morphology**. Oligodendrocytes grown in media containing thyroid hormone labeled for oligodendrocyte markers at day 3 *in vitro*. Oligodendrocytes did not label positively for the immature OPC markers NG2 (A) or A2B5 (B), but did label for the mature oligodendrocyte markers GalC (C) and MBP (D).

Oligodendrocytes grown continuously with thyroid hormone matured rapidly and were seen to have multiple processes by day 1 *in vitro*. Oligodendrocytes were labeled with antibodies to GalC and MBP on day 3 *in vitro *(Fig. 5C, D). Oligodendrocytes grown in thyroid hormone did not stain positively for NG2 or A2B5 (Fig. 5A,B) indicating that these oligodendrocytes were mature.

### Both "resting" and LPS-activated microglia reduce cell death of mature oligodendrocytes

**Figure 6:**
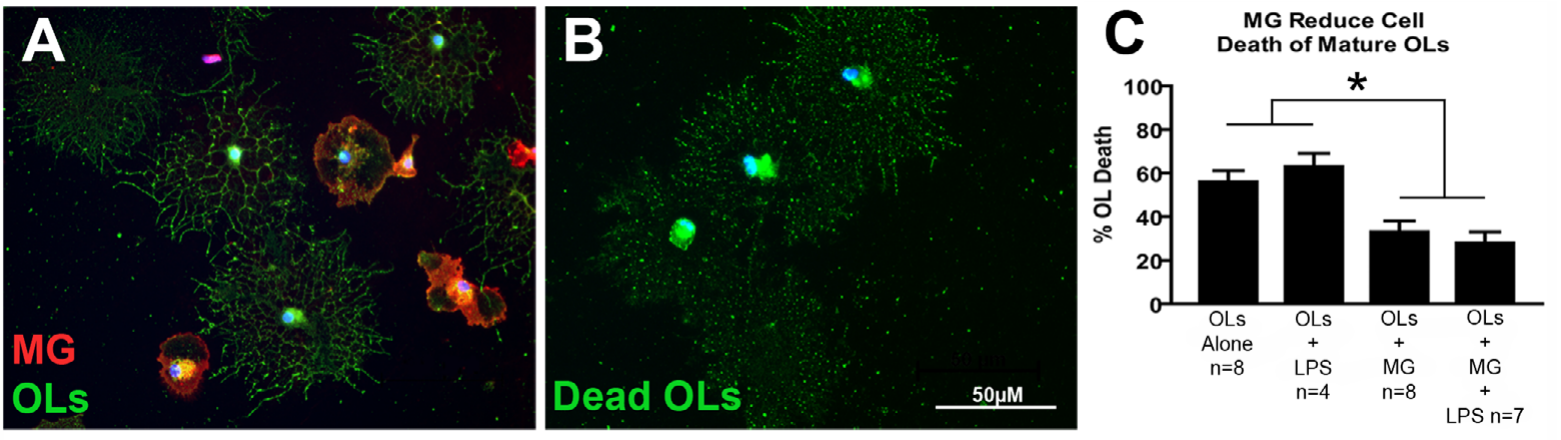
**Microglia and LPS-activated microglia both increase oligodendrocyte survival**. Oligodendrocytes were cultured in combination with microglia (A). Dead oligodendrocytes were visible by cellular debris left behind after fixation and staining (B) and therefore oligodendrocyte cell death was quantified as percent cell death based on cell counts. Both microglia and microglia activated by LPS significantly reduced the percentage of dead oligodendrocytes (C, * = p < 0.05, factorial ANOVA for main effect of microglia, error bars = SEM). The protective effect of microglia was unchanged by the presence of LPS (p < 0.05 factorial ANOVA).

For these experiments, pure oligodendrocyte cultures, with and without microglia added, were utilized (Fig. 6A). Oligodendrocyte death was quantified as percent dead out of total since remains of individual dead oligodendrocytes were still clearly visible after fixation and staining (Fig. 6B). As with OPCs, there was no main effect of 10 ng/ml LPS alone on oligodendrocyte survival. However, there was a main effect of microglia on oligodendrocyte survival (Fig. 6C, p < 0.001, factorial ANOVA). In the presence of microglia, oligodendrocyte death was significantly decreased to 33 ± 8% from control values of 56 ± 10% (p = 0.013, ANOVA, Tukey's post-hoc), and in the presence of microglia and LPS, oligodendrocyte death was significantly reduced to 28 ± 9% (p = 0.004, ANOVA, Tukey's post-hoc). Analysis of total number (live + dead) oligodendrocytes revealed no effect of treatment group on total oligodendrocyte number, verifying that microglia did not alter oligodendrocyte attachment to the culture substrate (data not shown).

Thyroid hormone has been shown to inhibit oligodendrocyte proliferation [49]. As expected, no oligodendrocytes in the control condition incorporated BrdU, and neither microglia nor LPS alone or in combination induced BrdU incorporation into oligodendrocytes (data not shown). This verifies that microglia mediated protection of oligodendrocytes was due to increased oligodendrocyte survival and not cell division.

To exclude a possible influence of thyroid hormone on the pro-survival effect of microglia, the experiment was repeated with thyroid hormone-containing media being replaced with thyroid hormone-free media on day 1 *in vitro *(at the same time microglia were added to oligodendrocytes). Under these conditions, the significant protective effect of microglia was retained (p = 0.043, factorial ANOVA, data not shown).

### Microglia reduce apoptosis of mature oligodendrocytes

**Figure 7:**
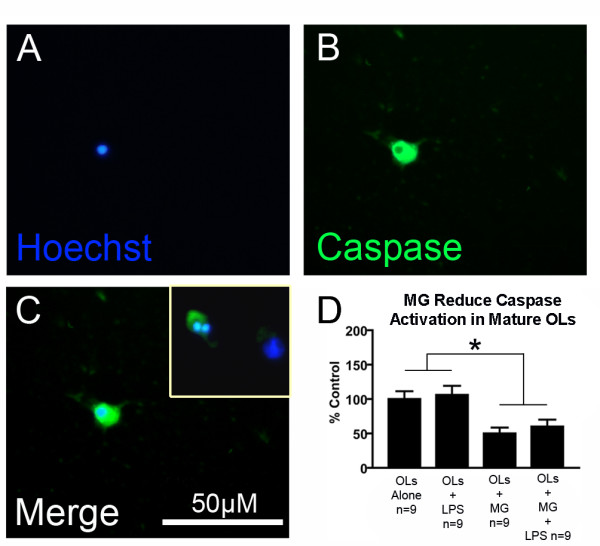
**Microglia reduce caspase activation in oligodendrocytes**. Fragmented and condensed nuclei in oligodendrocyte cultures (A) were found in all cell bodies that labeled with a caspase indicator probe (B), which allowed for identification of oligodendrocytes by morphology. Overlap of apoptotic-morphology nuclei and the caspase indicator were used to confirm apoptotic cell death (C). The inset in panel C shows another representative image of a fragmented nucleus in an oligodendrocyte positive for caspase activation. Cell counts were conducted to quantify caspase activation (D, * = p < 0.05, factorial ANOVA for main effect of microglia, error bars = SEM). Microglia and LPS-activated microglia both reduced oligodendrocyte apoptosis, suggesting that increased oligodendrocyte survival in the presence of microglia is due to a reduction in apoptosis. As in figure 5, the protective effect of microglia was unchanged by the presence of LPS (factorial ANOVA).

Since oligodendrocytes in our study were grown without growth factors such as CNTF, that have been shown to promote oligodendrocyte survival *in vitro *[50], we utilized a caspase activation assay to determine if oligodendrocytes underwent apoptosis over time *in vitro*. Previously, we have used specific activated caspase-3 antibodies in oligodendrocytes [44], however in this study we wished to examine a broad range of activated caspase enzymes. Using a poly-caspase detection assay, we evaluated oligodendrocyte apoptosis at various time points and found that oligodendrocyte apoptosis increased over time in culture (data not shown). Since caspase activation was highest at the terminal time point of 3 DIV, we conducted an experiment examining oligodendrocyte caspase activation with and without microglia and 10 ng/ml LPS at this time point. Apoptotic oligodendrocytes were seen to have condensed, pyknotic or fragmented nuclei (Fig. 7A) and labeled brightly with the caspase detector (Fig. 7B). The caspase probe labeling illuminated oligodendrocyte cell bodies so that it was possible to verify the cell phenotype. No microglia were observed to be labeled with the caspase probe. There was a significant reduction in oligodendrocyte caspase activation in the presence of microglia, regardless of the presence of LPS (Fig. 7C, p < 0.001, factorial ANOVA). In the presence of microglia, caspase activation was significantly decreased to 50 ± 9% of control values (p = 0.015), and in the presence of microglia and LPS, caspase activation was decreased to 60 ± 10% of control values, but did not achieve statistical significance (p = 0.064). The reduction of time-dependent caspase activation in oligodendrocytes in the presence of microglia likely explains the increase in oligodendrocyte survival in the presence of microglia and LPS-activated microglia (see Fig. 6).

### Microglia undergo cell death in response to LPS activation

**Figure 8:**
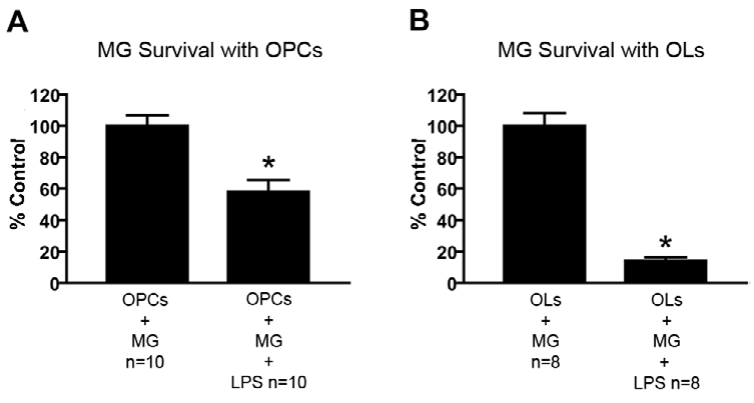
**Assessment of microglial cell death**. In experiments in which microglia were combined with OPCs, 10 ng/ml LPS significantly reduced microglia number to 58 ± 8% of control, based on cell counts (A). In experiments in which microglia were combined with oligodendrocytes, 10 ng/ml LPS significantly reduced microglia number to 14 ± 2% of control (B, * = p < 0.05, T-test, error bars = SEM).

Microglia survival was assessed after LPS activation in experiments where OPC and oligodendrocyte survival were measured. In experiments conducted with microglia in combined culture with OPCs, where media contained bFGF and PDGF, microglia survival was significantly decreased to 58 ± 8% of control values after treatment with 10 ng/ml LPS for 24 hours (p < 0.001, Student's T-test, Fig. 8A). In experiments conducted with microglia in culture with oligodendrocytes in media containing thyroid hormone, microglia numbers were significantly decreased to 14 ± 2% of control values after treatment with 10 ng/ml LPS for 24 hours (p < 0.001, Student's T-test, Fig. 8B).

To determine if activation-induced microglia death was due to the presence of OPCs or oligodendrocytes in culture with microglia, an experiment was conducted with microglia cultured alone. As in the previous experiment, microglia survival in both media compositions was significantly reduced in response to activation with 10 ng/ml LPS (data not shown). Thus, microglial cell death in response to LPS-activation was not due to the presence of OPCs or oligodendrocytes in culture. To confirm that lower numbers of microglia counted were due to microglial death and not simply detachment from coverslips before fixation, a LDH assay was used to assess microglial cell death in response to LPS in the aforementioned media compositions. 10 ng/ml LPS induced significant LDH release from microglia as compared to controls in both media compositions (data not shown). Therefore, lower microglia counts represent cell death and not simply detachment of microglia from the culture substrate.

## Discussion

Here we demonstrate that the maturation state of oligodendrocyte lineage cells has a profound effect on their response to microglia and microglial activation. While reducing the survival of OPCs, LPS-activated microglia increase the survival of mature oligodendrocytes. It is especially significant that in both cases, activated microglia were able to reverse the baseline state of oligodendrocyte lineage cells: normally proliferating OPCs were lost as a consequence of microglia activation, and mature oligodendrocytes that are normally undergoing apoptosis are rescued by activated microglia. A recent study has shown that as OPCs mature to oligodendrocytes a wide range of genes are differentially transcribed [47]. It is important to appreciate that even though OPCs are able to differentiate into mature oligodendrocytes in a matter of days both *in vitro *[47] and *in vivo *[51], these two cell types have very different properties and functional capabilities. Our data support the growing appreciation of the differences between these cells, showing that OPCs and mature oligodendrocytes could undergo divergent fates in an environment where activated microglia are present.

Several previous studies have shown that LPS-activated microglia are toxic to OPCs both *in vitro *and *in vivo *[23,24,48] and that this effect is mediated in part by oxidative stress [29]. Our studies indicate that mature oligodendrocytes benefit from the presence of microglia, and presumably resist the deleterious effects that activated microglia exert on OPCs. This is consistent with other studies showing oligodendrocytes to be more resistant than OPCs to a wide variety of insults, including TNFα [52], interferon gamma [53], excitotoxicity [54,55], radiation injury [56], and free radical mediated injury [57]. Many changes that occur in oligodendrocytes as they mature could promote resistance to injury, including a decrease in ionotropic glutamate receptor expression [58], an increase in metabotropic glutamate receptor expression [59], changes in pro- or anti-apoptotic gene expression [60], or increased antioxidant enzymes [61]. It is possible that mature oligodendrocytes in our study resisted microglia-mediated injury due to increased glutathione stores compared to OPCs, as reported by Fragoso and colleagues [62], which would render them less susceptible to microglia-mediated oxidative injury.

Though our data show that microglia only induced a trend toward increased OPC proliferation (Fig. 4), a previous report has shown that media from activated microglia have the ability to increase OPC proliferation [63]. The difference between this study and ours may lie in the different preparations used. While Filipovic and Zecevic [63] showed that microglia secrete factors that can induce OPC proliferation, this does not exclude the possibility that other microglia-derived factors can induce OPC cell death as well. If OPC death is induced by short-acting substances such as free radicals, and OPC proliferation is induced by more stable proteins, it is possible that conditioned medium from activated microglia, as used by Filipovic and Zecevic [63], would be more likely to isolate the proliferative effects of activated microglia. This is in contrast to our preparation where microglia and OPCs were in culture together and all substances produced by microglia had direct and immediate access to OPCs.

Understanding the physiologic differences between mature and immature oligodendrocytes may lead to therapeutic strategies for preserving OPCs vulnerable to loss in periventricular leukomalacia, or for preserving OPCs that proliferate after CNS injury. It has been shown previously that OPCs proliferate after SCI [64]. However, the net effect of SCI is to reduce oligodendrocyte numbers, at least in the long tracts damaged by the lesion [10]. Recent data from our laboratory suggest that some OPCs that proliferate after spinal cord injury die by apoptosis after the population initially expands (unpublished data). It is possible that proliferating OPCs *in vivo*, as in our *in vitro *study, are unable to withstand toxins produced by activated microglia after spinal cord injury. This OPC proliferation followed by apoptosis may represent a failed attempt at remyelination after spinal cord injury due to OPCs' intrinsic vulnerability to inflammation.

Though no previous study to our knowledge has directly compared the effects of activated microglia on different stages of oligodendrocyte lineage cells, some studies have previously examined microglia-mediated oligodendrocyte survival. Pro-survival factors secreted by microglia, such as insulin-like growth factor-2, may protect oligodendrocytes from TNFα and other molecules produced by activated microglia [33]. However, microglia have been shown to reverse their phenotype from protective to toxic for oligodendrocytes after activation with interferon-gamma [34]. The fact that in our experiments, LPS-activation did not change the anti-apoptotic effect of microglia, shows that increased activation does not always cause microglia to lose their protective effects. Additionally, recent data show that activated microglia can induce or hinder OPC proliferation depending on the cytokine profile used to activate microglia [36]. Recently, different pro-inflammatory substances that activate microglia have been shown to have different effects on demyelination and OPC proliferation *in vivo *[65]. In the injured CNS, multiple inflammatory cytokines are produced [66] and the combination of cytokines and other molecules that induce inflammation may change over time, potentially resulting in a dynamic microglia phenotype after injury. Further studies should be directed toward ascertaining how microglial activation changes over time and in response to different stimuli.

Microglia activated with LPS have been previously shown to undergo cell death in a dose-responsive manner [67]. As with microglia-mediated OPC death, microglial "autocrine" death can be mediated by free radicals [68]. LPS exposure can induce caspase activation in microglia [69], but LPS can also cause microglia to be more likely to undergo necrotic cell death under some conditions [70]. In our experiments, we did not observe caspase activation in microglia. This may be because microglial apoptosis occurred early after LPS addition, or that microglial cell death was primarily necrotic in nature. It is also possible that microglia detached from the growth substrate prior to undergoing cell death, as microglia fragments were rarely observed, unlike OPCs and oligodendrocytes. Regardless of the nature of microglial cell death, it is important to note that the pro-survival effect of microglia on oligodendrocytes was preserved even as microglia numbers were markedly reduced due to LPS activation. It is possible that sufficient microglia-derived survival factors for oligodendrocytes were produced before microglia were lost due to LPS activation. Alternately, the pro-survival effects of microglia may only require a small number of microglia, and the microglia that persist in the presence of LPS-activation may be sufficient to support oligodendrocyte survival. In either case, the ability of a reduced population of microglia to maintain their pro-survival effect on oligodendrocytes demonstrates that microglial activation is not necessarily harmful to all CNS cells. Microglial cell death also occurs during neuroinflammation and after injury *in vivo *[13,71] and may be a form of autoregulation to limit the microglial response to injury and reduce damage to other CNS cells.

## Conclusion

In conclusion, we demonstrate that activated microglia decrease survival of OPCs but that microglia, whether or not they have been stimulated with LPS, reduce apoptosis and increase survival of mature oligodendrocytes *in vitro*. The effects of activated microglia on OPC and oligodendrocyte viability are present even as microglia themselves undergo cell death due to activation, indicating that the effects of microglia on OPC and oligodendrocyte survival may not require a stable population of microglia. A further understanding of the mechanisms behind OPCs' and oligodendrocytes' differential response to microglia may aid in the development of therapies for several CNS diseases where microglial activation and OPC and oligodendrocyte death occur together.

## Competing interests

The author(s) declare that they have no competing interests.

## Authors' contributions

BAM designed and carried out cell culture experiments, analyzed data and prepared the manuscript and figures. JMC assisted with experimental design and cell culture. CAT assisted with experimental design and cell culture and planned and carried out TNFα ELISAs. ARF assisted with experimental design, statistical analysis and manuscript preparation. JCB and MSB planned experiments, interpreted data, and participated in manuscript preparation. All authors have read and approved the contents of the final manuscript.

## References

[B1] Raff MC, Miller RH, Noble M (1983). A glial progenitor cell that develops in vitro into an astrocyte or an oligodendrocyte depending on culture medium. Nature.

[B2] Wolswijk G, Noble M (1989). Identification of an adult-specific glial progenitor cell. Development.

[B3] Scolding NJ, Rayner PJ, Compston DA (1999). Identification of A2B5-positive putative oligodendrocyte progenitor cells and A2B5-positive astrocytes in adult human white matter. Neuroscience.

[B4] McTigue DM, Horner PJ, Stokes BT, Gage FH (1998). Neurotrophin-3 and brain-derived neurotrophic factor induce oligodendrocyte proliferation and myelination of regenerating axons in the contused adult rat spinal cord. J Neurosci.

[B5] Sun F, Lin C, McTigue DM, Shan X, C.A. T, J.C. B, Beattie MS (2006). Dorsal rhizotomy induces oligodendrocyte progenitor proliferation and oligodendrocyte genesis, while spinal contusion induces oligodendrocyte death. J Neurosci.

[B6] Woodruff RH, Fruttiger M, Richardson WD, Franklin RJ (2004). Platelet-derived growth factor regulates oligodendrocyte progenitor numbers in adult CNS and their response following CNS demyelination. Mol Cell Neurosci.

[B7] Blumenthal I (2004). Periventricular leucomalacia: a review. Eur J Pediatr.

[B8] Haynes RL, Folkerth RD, Keefe RJ, Sung I, Swzeda LI, Rosenberg PA, Volpe JJ, Kinney HC (2003). Nitrosative and oxidative injury to premyelinating oligodendrocytes in periventricular leukomalacia. J Neuropathol Exp Neurol.

[B9] Anderson DW, Ellenberg JH, Leventhal CM, Reingold SC, Rodriguez M, Silberberg DH (1992). Revised estimate of the prevalence of multiple sclerosis in the United States. Ann Neurol.

[B10] Beattie MS, Hermann GE, Rogers RC, Bresnahan JC (2002). Cell death in models of spinal cord injury. Prog Brain Res.

[B11] Castejon OJ (1985). Electron microscopic study of central axons degeneration in traumatic human brain edema. J Submicrosc Cytol.

[B12] Crowe MJ, Bresnahan JC, Shuman SL, Masters JN, Beattie MS (1997). Apoptosis and delayed degeneration after spinal cord injury in rats and monkeys. Nat Med.

[B13] Shuman SL, Bresnahan JC, Beattie MS (1997). Apoptosis of microglia and oligodendrocytes after spinal cord contusion in rats. J Neurosci Res.

[B14] Hess DC, Abe T, Hill WD, Studdard AM, Carothers J, Masuya M, Fleming PA, Drake CJ, Ogawa M (2004). Hematopoietic origin of microglial and perivascular cells in brain. Exp Neurol.

[B15] Streit WJ, Graeber MB, Kreutzberg GW (1988). Functional plasticity of microglia: a review. Glia.

[B16] Kreutzberg GW (1996). Microglia: a sensor for pathological events in the CNS. Trends Neurosci.

[B17] Carbonell WS, Murase S, Horwitz AF, Mandell JW (2005). Migration of perilesional microglia after focal brain injury and modulation by CC chemokine receptor 5: an in situ time-lapse confocal imaging study. J Neurosci.

[B18] Dasgupta S, Jana M, Liu X, Pahan K (2003). Role of very-late antigen-4 (VLA-4) in myelin basic protein-primed T cell contact-induced expression of proinflammatory cytokines in microglial cells. J Biol Chem.

[B19] Jana M, Dasgupta S, Saha RN, Liu X, Pahan K (2003). Induction of tumor necrosis factor-alpha (TNF-alpha) by interleukin-12 p40 monomer and homodimer in microglia and macrophages. J Neurochem.

[B20] Selmaj KW, Raine CS (1988). Tumor necrosis factor mediates myelin and oligodendrocyte damage in vitro. Ann Neurol.

[B21] Nakamura Y, Ohmaki M, Murakami K, Yoneda Y (2003). Involvement of protein kinase C in glutamate release from cultured microglia. Brain Res.

[B22] Benveniste EN (1997). Role of macrophages/microglia in multiple sclerosis and experimental allergic encephalomyelitis. J Mol Med.

[B23] Pang Y, Cai Z, Rhodes PG (2000). Effects of lipopolysaccharide on oligodendrocyte progenitor cells are mediated by astrocytes and microglia. J Neurosci Res.

[B24] Pang Y, Cai Z, Rhodes PG (2003). Disturbance of oligodendrocyte development, hypomyelination and white matter injury in the neonatal rat brain after intracerebral injection of lipopolysaccharide. Brain Res Dev Brain Res.

[B25] Sriram S, Rodriguez M (1997). Indictment of the microglia as the villain in multiple sclerosis. Neurology.

[B26] Christensen RN, Ha BK, Sun F, Bresnahan JC, Beattie MS (2006). Kainate induces rapid redistribution of the actin cytoskeleton in ameboid microglia. J Neurosci Res.

[B27] Noda M, Nakanishi H, Nabekura J, Akaike N (2000). AMPA-kainate subtypes of glutamate receptor in rat cerebral microglia. J Neurosci.

[B28] Thery C, Mallat M (1993). Influence of interleukin-1 and tumor necrosis factor alpha on the growth of microglial cells in primary cultures of mouse cerebral cortex: involvement of colony-stimulating factor 1. Neurosci Lett.

[B29] Li J, Baud O, Vartanian T, Volpe JJ, Rosenberg PA (2005). Peroxynitrite generated by inducible nitric oxide synthase and NADPH oxidase mediates microglial toxicity to oligodendrocytes. Proc Natl Acad Sci U S A.

[B30] Zajicek JP, Wing M, Scolding NJ, Compston DA (1992). Interactions between oligodendrocytes and microglia. A major role for complement and tumour necrosis factor in oligodendrocyte adherence and killing. Brain.

[B31] Filipovic R, Zecevic N (2005). Interaction between Microglia and Oligodendrocyte Cell Progenitors Involves Golli Proteins. Ann N Y Acad Sci.

[B32] Shaked I, Tchoresh D, Gersner R, Meiri G, Mordechai S, Xiao X, Hart RP, Schwartz M (2005). Protective autoimmunity: interferon-gamma enables microglia to remove glutamate without evoking inflammatory mediators. J Neurochem.

[B33] Nicholas RS, Stevens S, Wing MG, Compston DA (2002). Microglia-derived IGF-2 prevents TNFalpha induced death of mature oligodendrocytes in vitro. J Neuroimmunol.

[B34] Nicholas R, Stevens S, Wing M, Compston A (2003). Oligodendroglial-derived stress signals recruit microglia in vitro. Neuroreport.

[B35] Arnett HA, Mason J, Marino M, Suzuki K, Matsushima GK, Ting JP (2001). TNF alpha promotes proliferation of oligodendrocyte progenitors and remyelination. Nat Neurosci.

[B36] Butovsky O, Landa G, Kunis G, Ziv Y, Avidan H, Greenberg N, Schwartz A, Smirnov I, Pollack A, Jung S, Schwartz M (2006). Induction and blockage of oligodendrogenesis by differently activated microglia in an animal model of multiple sclerosis. J Clin Invest.

[B37] Popovich PG, Guan Z, McGaughy V, Fisher L, Hickey WF, Basso DM (2002). The neuropathological and behavioral consequences of intraspinal microglial/macrophage activation. J Neuropathol Exp Neurol.

[B38] Ryu JK, Nagai A, Kim J, Lee MC, McLarnon JG, Kim SU (2003). Microglial activation and cell death induced by the mitochondrial toxin 3-nitropropionic acid: in vitro and in vivo studies. Neurobiol Dis.

[B39] Zucconi GG, Laurenzi MA, Semprevivo M, Torni F, Lindgren JA, Marinucci E (2002). Microglia activation and cell death in response to diethyl-dithiocarbamate acute administration. J Comp Neurol.

[B40] Gehrmann J, Banati RB (1995). Microglial turnover in the injured CNS: activated microglia undergo delayed DNA fragmentation following peripheral nerve injury. J Neuropathol Exp Neurol.

[B41] Lee SJ, Lee S (2002). Toll-like receptors and inflammation in the CNS. Curr Drug Targets Inflamm Allergy.

[B42] Kim DC, Kim SH, Jeong MW, Baek NI, Kim KT (2005). Effect of rottlerin, a PKC-delta inhibitor, on TLR-4-dependent activation of murine microglia. Biochem Biophys Res Commun.

[B43] McCarthy KD, de Vellis J (1980). Preparation of separate astroglial and oligodendroglial cell cultures from rat cerebral tissue. J Cell Biol.

[B44] Miller BA, Sun F, Christensen RN, Ferguson AR, Bresnahan JC, Beattie MS (2005). A sublethal dose of TNFalpha potentiates kainate-induced excitotoxicity in optic nerve oligodendrocytes. Neurochem Res.

[B45] Grabarek J, Amstad P, Darzynkiewicz Z (2002). Use of fluorescently labeled caspase inhibitors as affinity labels to detect activated caspases. Hum Cell.

[B46] Kondo T, Raff M (2000). The Id4 HLH protein and the timing of oligodendrocyte differentiation. EMBO J.

[B47] Dugas JC, Tai YC, Speed TP, Ngai J, Barres BA (2006). Functional genomic analysis of oligodendrocyte differentiation. J Neurosci.

[B48] Lehnardt S, Lachance C, Patrizi S, Lefebvre S, Follett PL, Jensen FE, Rosenberg PA, Volpe JJ, Vartanian T (2002). The toll-like receptor TLR4 is necessary for lipopolysaccharide-induced oligodendrocyte injury in the CNS. J Neurosci.

[B49] Baas D, Bourbeau D, Sarlieve LL, Ittel ME, Dussault JH, Puymirat J (1997). Oligodendrocyte maturation and progenitor cell proliferation are independently regulated by thyroid hormone. Glia.

[B50] Louis JC, Magal E, Takayama S, Varon S (1993). CNTF protection of oligodendrocytes against natural and tumor necrosis factor-induced death. Science.

[B51] Tripathi R, McTigue DM (2007). Prominent oligodendrocyte genesis along the border of spinal contusion lesions. Glia.

[B52] Pang Y, Cai Z, Rhodes PG (2005). Effect of tumor necrosis factor-alpha on developing optic nerve oligodendrocytes in culture. J Neurosci Res.

[B53] Baerwald KD, Popko B (1998). Developing and mature oligodendrocytes respond differently to the immune cytokine interferon-gamma. J Neurosci Res.

[B54] Rosenberg PA, Dai W, Gan XD, Ali S, Fu J, Back SA, Sanchez RM, Segal MM, Follett PL, Jensen FE, Volpe JJ (2003). Mature myelin basic protein-expressing oligodendrocytes are insensitive to kainate toxicity. J Neurosci Res.

[B55] Wosik K, Ruffini F, Almazan G, Olivier A, Nalbantoglu J, Antel JP (2004). Resistance of human adult oligodendrocytes to AMPA/kainate receptor-mediated glutamate injury. Brain.

[B56] Fukuda A, Fukuda H, Swanpalmer J, Hertzman S, Lannering B, Marky I, Bjork-Eriksson T, Blomgren K (2005). Age-dependent sensitivity of the developing brain to irradiation is correlated with the number and vulnerability of progenitor cells. J Neurochem.

[B57] Bernardo A, Greco A, Levi G, Minghetti L (2003). Differential lipid peroxidation, Mn superoxide, and bcl-2 expression contribute to the maturation-dependent vulnerability of oligodendrocytes to oxidative stress. J Neuropathol Exp Neurol.

[B58] Itoh T, Beesley J, Itoh A, Cohen AS, Kavanaugh B, Coulter DA, Grinspan JB, Pleasure D (2002). AMPA glutamate receptor-mediated calcium signaling is transiently enhanced during development of oligodendrocytes. J Neurochem.

[B59] Luyt K, Varadi A, Durant CF, Molnar E (2006). Oligodendroglial metabotropic glutamate receptors are developmentally regulated and involved in the prevention of apoptosis. J Neurochem.

[B60] Itoh T, Itoh A, Pleasure D (2003). Bcl-2-related protein family gene expression during oligodendroglial differentiation. J Neurochem.

[B61] Folkerth RD, Haynes RL, Borenstein NS, Belliveau RA, Trachtenberg F, Rosenberg PA, Volpe JJ, Kinney HC (2004). Developmental lag in superoxide dismutases relative to other antioxidant enzymes in premyelinated human telencephalic white matter. J Neuropathol Exp Neurol.

[B62] Fragoso G, Martinez-Bermudez AK, Liu HN, Khorchid A, Chemtob S, Mushynski WE, Almazan G (2004). Developmental differences in HO-induced oligodendrocyte cell death: role of glutathione, mitogen-activated protein kinases and caspase 3. J Neurochem.

[B63] Filipovic R, Zecevic N (2005). Lipopolysaccharide affects Golli expression and promotes proliferation of oligodendrocyte progenitors. Glia.

[B64] McTigue DM, Wei P, Stokes BT (2001). Proliferation of NG2-positive cells and altered oligodendrocyte numbers in the contused rat spinal cord. J Neurosci.

[B65] Schonberg DL, Popovich PG, McTigue DM (2007). Distinct intraspinal macorphage activation protocols differentially influence oligodendrocyte genesis. J Neurotrauma.

[B66] Yang L, Jones NR, Blumbergs PC, Van Den HC, Moore EJ, Manavis J, Sarvestani GT, Ghabriel MN (2005). Severity-dependent expression of pro-inflammatory cytokines in traumatic spinal cord injury in the rat. J Clin Neurosci.

[B67] Liu B, Wang K, Gao HM, Mandavilli B, Wang JY, Hong JS (2001). Molecular consequences of activated microglia in the brain: overactivation induces apoptosis. J Neurochem.

[B68] Lee P, Lee J, Kim S, Lee MS, Yagita H, Kim SY, Kim H, Suk K (2001). NO as an autocrine mediator in the apoptosis of activated microglial cells: correlation between activation and apoptosis of microglial cells. Brain Res.

[B69] Lee J, Hur J, Lee P, Kim JY, Cho N, Kim SY, Kim H, Lee MS, Suk K (2001). Dual role of inflammatory stimuli in activation-induced cell death of mouse microglial cells. Initiation of two separate apoptotic pathways via induction of interferon regulatory factor-1 and caspase-11. J Biol Chem.

[B70] Nagano T, Kimura SH, Takai E, Matsuda T, Takemura M (2006). Lipopolysaccharide sensitizes microglia toward Ca(2+)-induced cell death: mode of cell death shifts from apoptosis to necrosis. Glia.

[B71] White CA, McCombe PA, Pender MP (1998). Microglia are more susceptible than macrophages to apoptosis in the central nervous system in experimental autoimmune encephalomyelitis through a mechanism not involving Fas (CD95). Int Immunol.

